# *P. aeruginosa* Mediated Necroptosis in Mouse Tumor Cells Induces Long-Lasting Systemic Antitumor Immunity

**DOI:** 10.3389/fonc.2020.610651

**Published:** 2021-02-12

**Authors:** Jia-long Qi, Jin-rong He, Shu-mei Jin, Xu Yang, Hong-mei Bai, Cun-bao Liu, Yan-bing Ma

**Affiliations:** ^1^ Institute of Medical Biology, Chinese Academy of Medical Sciences and Peking Union Medical College, Kunming, China; ^2^ Institute of Medical Biology, Kunming Medical University, Kunming, China; ^3^ Department of Pathology, Yunnan Institute of Materia, Kunming, China

**Keywords:** dying tumor cell, tumor microenvironment, antitumor immunity, necroptosis, *P. aeruginosa*

## Abstract

Necroptosis is a form of programmed cell death (PCD) characterized by RIP3 mediated MLKL activation and increased membrane permeability *via* MLKL oligomerization. Tumor cell immunogenic cell death (ICD) has been considered to be essential for the anti-tumor response, which is associated with DC recruitment, activation, and maturation. In this study, we found that *P. aeruginosa* showed its potential to suppress tumor growth and enable long-lasting anti-tumor immunity *in vivo*. What’s more, phosphorylation- RIP3 and MLKL activation induced by *P. aeruginosa* infection resulted in tumor cell necrotic cell death and HMGB1 production, indicating that *P. aeruginosa* can cause immunogenic cell death. The necrotic cell death can further drive a robust anti-tumor response *via* promoting tumor cell death, inhibiting tumor cell proliferation, and modulating systemic immune responses and local immune microenvironment in tumor. Moreover, dying tumor cells killed by *P. aeruginosa* can catalyze DC maturation, which enhanced the antigen-presenting ability of DC cells. These findings demonstrate that *P. aeruginosa* can induce immunogenic cell death and trigger a robust long-lasting anti-tumor response along with reshaping tumor microenvironment.

**Graphical Abstract:**
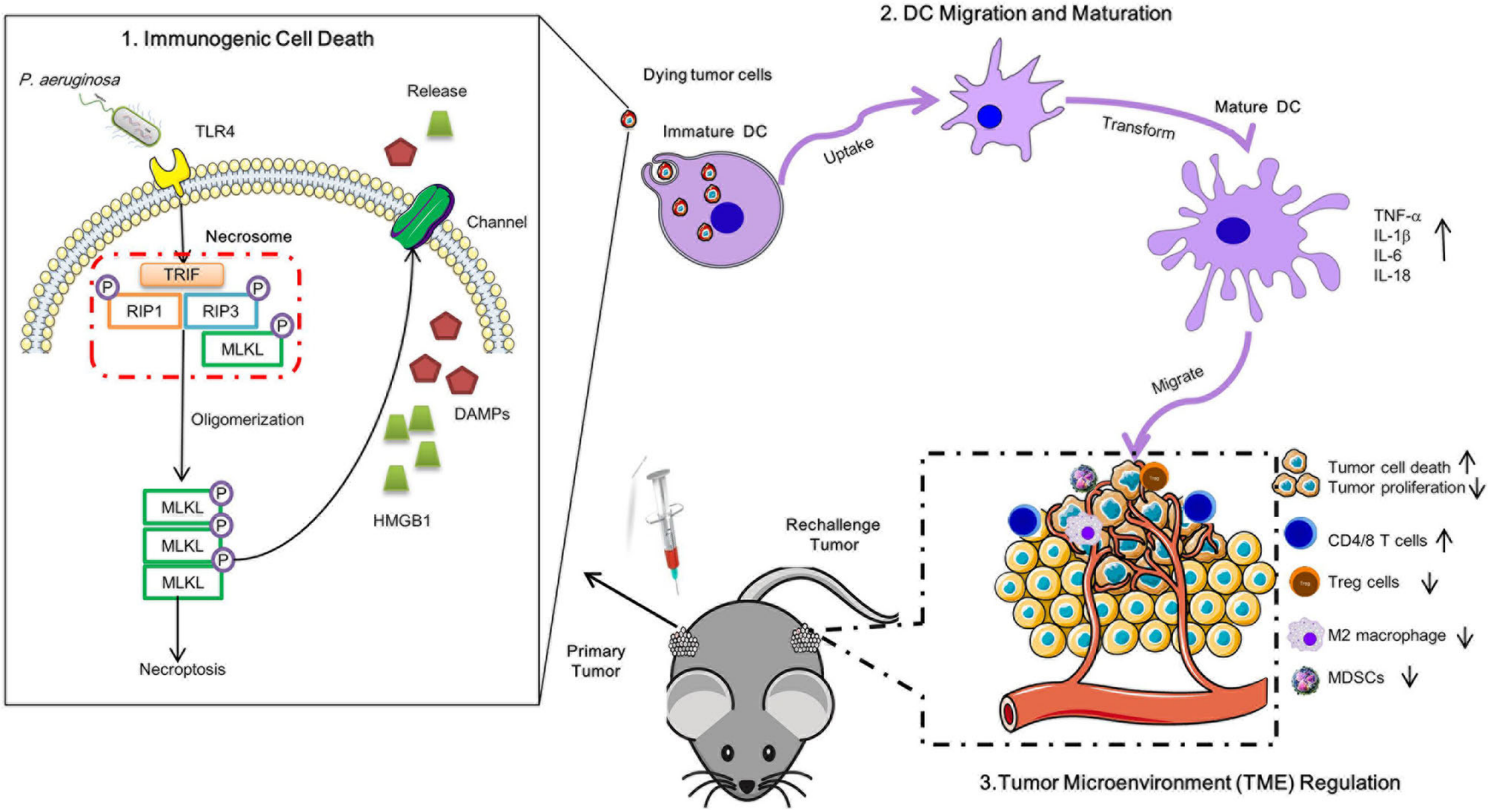
Schematic diagram of *P. aeruginosa* mediated anti-tumor immunity. A single low dose of *P. aeruginosa* injection not only curbed the growth of treated tumors *in situ* but also delayed the proliferation of distant tumors. *P. aeruginosa* active PANoptosis signaling pathways and triggered tumor cells death in a TLR4 dependent manner *in vitro*. Meanwhile, the necrotic-like dying tumor cells release HMGB1 matured DC cells, resulting in the reshaped tumor microenvironment in anti-tumor immunity.

## Highlights

1. *P. aeruginosa* infection triggered necrotic tumor cell death to enhance long-lasting anti-tumor response *in vivo*.
2. *P. aeruginosa*-induced robust antitumor immunity modulated tumor microenvironment (TME) immune cells infiltration and cytokines production.
3. Dying tumor cells orchestrated DC recruitment, activation, and maturation.

## Introduction

In the last decades, more research has revealed the potential connection between specific microorganisms and different types of cancer, which was observed by investigation microbiome in cancer patients (1). Microbiota can not only promote tumor cell death but also reshape tumor microenvironment (TME) to turn “cold” tumor into “hot” tumor by increasing the number of immune-effective cells and enhancing the anti-tumor ability, while decreasing the number of immune-suppressive cells and suppressing their tumor-suppressive function (2). Alternatively, bacteria-derived metabolites cause cancer cell autophagy and facilitate anti-tumor immune-surveillance (3). To this end, the bacteria-mediated anti-cancer effects can prominently affect the outcomes of immunotherapy.

The first immunotherapeutic bacteria applied to cancer immunotherapy can be dated back to the late 19th century (4). Up to now, numerous types of bacteria have been identified with robust ability to inhibit tumor growth with low toxicity (5). Although the most well-established studies of the relationship between the microbiota, tumorigenesis, and tumor drug-resistance have been focused on the gut microbiome, the acquisition of the human upper respiratory tract, especially lung microbiome, also plays a crucial role in protecting the host from infection, inflammation, and anti-tumor response (6, 7). The migration, maturation, and activation of innate immune cells in lung tissues require commensal host-microbial interaction.

*Pseudomonas aeruginosa* parasitized on the skin is an opportunistic Gram-negative pathogen that can also cause diverse infections in the lungs (8). Previous studies have demonstrated that the lipopolysaccharide of *P. aeruginosa* is essential to inhibit tumor growth as an adjuvant and promote interferon cytokines production (9). In addition, *P. aeruginosa*–mannose-sensitive hemagglutinin (PA-MSHA) enhances anti-tumor immunity of cord-blood cytokine-induced killer (CB-CIK) cells *via* IFN-γ release and Toll-like receptor (TLR) activation (10). What’s more, PA-MSHA directly stimulates T cell, migration, and maturation of dendritic cells (DC) *via* TLR4-dependent manner that elicits antitumor immunity (11). PA-MSHA also directly triggers tumor cell apoptosis, arrests cell cycle, as well as inhibits autophagy *via* EGFR pathway and/or p62 regulating (12–15). However, other types of cell death that can be triggered by *P. aeruginosa*, like necroptosis, in antitumor responses have not been comprehensively evaluated. Here, we employed a TC-1 solid tumor model to evaluate the role of *P. aeruginosa* triggered necroptosis in anti-tumor response.

Necroptosis is a form of programmed cell death that is characterized by RIP3 mediated MLKL activation and can increase cell membrane permeability by MLKL oligomerization (16). Necroptosis also participates in cardiovascular diseases (17), anti-bacteria (18), anti-viral infection (19), and even anti-tumor response (20). It has been reported that Gram-positive bacteria *S. aureus*-induce lung cancer cell A549 cell death is enhanced by TNF-α, which is associated by RIP3-dependented necroptosis (21). RIP3-mediated CXCL1 promote tumor associated macrophages (TAM)-induced adaptive immune suppression and myeloid-derived suppressor cells (MDSC)-induced adaptive immune suppression in pancreatic ductal adenocarcinoma (PDA) and colitis-associated cancer (CAC) (22, 23). Similarly, MLKL suppresses colon inflammation and tumorigenesis *via* MEK/ERK activation in DC (24). Intra-tumor therapy with an MLKL-mRNA-based vaccine inhibit CAC growth reshaped the tumor microenvironment inducing systemic antitumor response directed against neo-epitopes (25, 26). In addition, tumor cell immunogenic cell death (ICD) has been considered to be essential for anti-tumor response associated with DC recruitment, activation, and maturation (27, 28). Although chemotherapeutics normally trigger cell death in RIP3-/- or MLKL-/- tumor cell death *via* caspase-3-dependent apoptosis, it is unable to induce systemic anti-tumor response and inhibit tumor growth *in vivo*, which indicates necroptosis pathway plays a potential role in ICD (29). Notably, the danger-associated molecular patterns (DAMPs) released by necrotic cell death contribute to hosting immune system activation, particularly HMGB-1 released by the dying cell, which can stimulate DC maturation through TLR4-myD88 pathway (30). However, it is not clear whether bacteria-mediated necrotic cell death is a form of ICD and whether these bacteria-triggered dying cells can induce systemic anti-tumor immunity.

Herein, we identify a beneficial immunotherapeutic role of *P. aeruginosa* that mediates necrotic tumor cell death *in vitro* and *in vivo*, which provides a long-lasting anti-tumor immunity by reshaping systemic and tumor microenvironment. Surprisingly, *P. aeruginosa* not only inhibited tumor growth *in vivo* but also activated functions of DC cells, therefore indicating a strategy for designing bacteria-based immunotherapy against the tumor. These beneficial effects occur after co-administration of bacteria in tumor cells simultaneously, indicating that the protective effects are due to bacteria triggered RIP3-MLKL-dependent necroptosis. In conclusion, our findings demonstrate that *P. aeruginosa* triggers tumor necrotic cell death to promote robust antitumor immunity.

## Materials and Methods

### Ethics Statement

The Ethics Committee of Animal Care and Welfare of IMBCAMS and PUMC (Permit Number: SYXK (dian)2010-0007) approved all of the animal experiments according to the guidelines of the Institute of the Medical Biology Chinese Academy of Medical Sciences (IMBCAMS) & Perking Union Medical College (PUMC). All efforts were made to minimize animal suffering.

### Mice

All mice, six- to eight-week-old female C57BL/6N were purchased from Beijing Vital River Laboratory Animal Technology Co., Ltd. (Beijing, People’s Republic of China), maintained under specific pathogen-free (SPF) conditions and raised in the central animal care services of IMBCAMS. Before bacterial infection or tumor injection, mice were anesthetized with 3% isoflurane in oxygen for 2–5 min until immobile. At the experiment endpoints, mice were first anesthetized and then sacrificed by an increasing CO_2_ concentration for 5–10 min in a closed chamber until mice pupil dilation, stopped breathing, and stopped heartbeat.

### Bacteria Culture and Mice Infection

The clinical isolate *P. aeruginosa 1409*, which was identified and stored by our own lab, was grown overnight with shaking Luria-Bertani (LB) medium under 37°C. The next day, the bacteria were sub-cultured with fresh LB medium for 4 h. Until the bacterial concentration corresponding to an OD value reaches 0.5, the culture was collected with a centrifuge at 7000 × g for 10 min and washed the bacterial twice with sterile phosphate-buffered saline (PBS). The murine bacteremia model was established with a subcutaneous injection with a 50 μl per mouse total volume solution containing 3 × 10^6^ CFU to 3 × 10^8^ CFU bacterial in Basement Membrane Matrix (BD Bioscience, San Jose, CA, USA). Each group contains 8 mice and continuous monitoring of the bacterial challenged mice survival rate for seven days, considered as the endpoint.

### Tumor Model

To establish the tumor cell-grafted model, indicated 1 × 10^6^ cells incubated with- or without- 3 × 10^6^ CFU bacteria were mixed with Basement Membrane Matrix (BD Bioscience, San Jose, CA, USA) and subcutaneously implanted in the left dorsal flank of C57BL/6N mice for the primary tumor model construction. The mice were monitored three times per week for tumor growth using a slide caliper as described previously. Body weight changes were monitored every two days of tumor-bearing mice to identify the bacteria infection induced disadvantages for 15 days, and the survival rates were monitored until all mice were dead in the control group, and then the experimental group were euthanized. After the primary tumor established, tumor-free mice were rechallenged with TC-1 tumor cells in the other side flank of mice (n=5–6). Secondary tumor growth curve was monitored. All tumor volumes were measured with a caliper and calculated as (length × weidth^2^)/2.

### Indirect Immunofluorescence and H & E Staining

At the experiment endpoint, the mice were anesthetized and sacrificed. Tumor tissues were stripped and obtained, frozen in liquid nitrogen, embedded in optimal cutting temperature compound (OCT), and sectioned in 5 µm thickness pieces. The sections were performed for indirect immunofluorescence staining assay. Briefly, the cryo-sections were fixed in 95% alcohol and permeabilized with 2% BSA in 0.1% TritonX-100/PBS overnight at 4°C for 30 min, and then blocked with 5% bovine serum albumin (BSA) for 1 h. After blocking, the sections were incubated with Caspase-3, MLKL, MPO, and Ki67 primary antibody overnight at 4°C, respectively. The bound antibodies were labeled with FITC fluorescent secondary antibodies for 1 h at room temperature. After washing, all cryo-sections were stained with 4′,6-diamidino-2-phenylindole (DAPI) for 15 min at room temperature, and then photographed under a fluorescence microscope. Tumor tissues frozen sections were also stained for histologic examination by staining with hematoxylin and eosin (H & E) kit, according to the manufacturer’s protocol. Areas of positive were measured with software Image J.

### Flow Cytometry Assay (FCM)

Lymphocytes cells isolated from the spleen and tumor tissues of tumor-bearing mice were plated into a 96-microwells plate for immune cell profiles assay. Cell surface staining was performed by incubating cells with corresponding antibodies for 20 min on ice. Cells were stained with APC labeled anti mouse CD3 and FITC labeled anti mouse CD4 for CD4 positive T cells; APC labeled anti mouse CD3 and FITC labeled anti mouse CD8 for CD8 positive T cells; APC labeled anti mouse CD3 and PE labeled anti mouse NK1.1 for NK and NKT cells; APC labeled anti mouse CD11b and FITC labeled F4/80 for macrophages; APC labeled anti mouse CD11b and PE labeled Gr-1 for MDSC cells; FITC labeled anti mouse CD4, PE labeled anti mouse CD25 and APC labeled anti mouse Foxp3 for Treg cells. All FCM reagents were purchased from Biolegend, San Diego, CA, USA. After washing, the cells were resuspended in 200 µl staining buffer and analyzed by flow cytometry (CytoFLEX LX, Beckman, USA), and the data were analyzed using CytExpert 2.0 software.

### Enzyme-Linked Immunospot Assay (ELISPOT)

For ELISPOT assay, the protocol was the same as previously reported. In brief, splenic lymphocytes and tumor-infiltrating lymphocytes were harvested and extracted by mouse lymphocytes separation kit (BioLedgen, USA), and then washed with fresh RPMI 1640 medium. Next, 1 × 10^5^ cells were seeded into a 96-microwells plate, which was pre-coated with IFN-γ- and IL-2-specific coated antibodies, and then 2 ug/ml HPV 16 E7_49-57_ specific peptides was added into the plates. After 18 h stimulation, the culture medium was gently discarded and fresh pre-cooled water was added for rupturing lymphocytes. Then, incubating IFN-γ- and IL-2- capture secondary antibodies that were labeled with biotin for 1 h at room temperature, followed by avidin-conjugated HRP. After immuno-imaging, spots representing activated lymphocytes were counted by an ELISPOT reader system (AID Diagnostika GmbH, Strabberg, Germany).

### LDH Release Assay

Tumor cells were stimulated with 10 MOI *P. aeruginosa 1409* for 12 h, cell culture supernatants were gently discarded and incubated with LDH release regent for 1 h, all supernatants were removed to a new cell culture plate and 60 μl LDH working solution was added and then slowly sharking at room temperature for 30 min in the dark environment. Absorbance [A490nm-A620 nm] stands for the quantification of LDH release.

### Immunoblotting Assay

For immunoblot assay, TC-1 cells were seeded into 12-well plates, 10^6^ cells per well; the next day before infection, cells were washed with PBS three times and then 500 μl FBS- and antibiotics-free RPMI1640 medium added to each well. To evaluate bacterial infection on TC-1, 10 MOI *P. aeruginosa 1409* were added into cell culture for 1 h, 4 h, and 12 h at 37°C in 5% CO2 atmosphere. All cells were washed with PBS three times and lysed in 100 μl RIPA (Tris pH 7.4 50 mmol/L, NaCl 150 mmol/L, NP-40 1%, SDS 0.1%, EDTA 2 mmol/L) buffer containing proteinase and phosphatase cocktail inhibitor. All samples were separated by 12% SDS-PAGE and transfered to polyvinylidene fluoride membranes (PVDF), which were blocked by 5% BSA blocking reagent at room temperature for 2 h. The primary antibodies used: anti-MLKL (CST, 37705), anti-pMLKL (CST, 37333S), anti-RIP3 (CST, 95702), anti-pRIP3 (CST, 57220), anti-LC3B (Abcam; ab51520), anti-HMGB1 (Abcam; ab 18256), anti-β-actin (Abcam; ab6276), anti-GAPDH. The secondary antibodies used HRP-labeled anti-rabbit or mouse antibodies (Abcam). At last, the blotting was developed by the ECL chemiluminescence substrate. Image J software was used to quantify the Western Blot bands grayscale value.

### ELISA

The DCs were incubated with bacteria-treated dying tumor cells for 24 h. The cell culture mediums were collected for cytokines quantitation. The IL-1β, IL-6, TNF-α, and MCP-1 were measured by mouse IL-1β, IL-6, TNF-α, and MCP-1 quantikine ELISA kits (R&D systems), respectively, according to the manufacturer’s protocol.

### Real-Time Quantitative PCR

Indicated 1 × 10^6^ TC-1 tumor cells were seeded into a 12-well cell culture plates. The next day, 1 × 10^7^ CFU bacteria were added into the cell culture for stimulation for 1 h and 4 h, the medium were discarded, and the dying tumor cells were harvest. Total RNA was isolated using TRIzol (RNAiso, Takara) and purified by the chloroform-phenol extraction kits. cDNA was reverse transcribed with the SureScript First-stand cDNA Synthesis Kit (GeneCopoeia) and then detected by the All-in-One™ miRNA qRT-PCR Detection Kit (GeneCopoeia). Real-time quantitative PCR (RT-qPCR) was performed on a Bio-Rad CFX-96 Touch Real-Time Detection system. Primer sequences are listed in supplemental **Table S1**.

### Statistical Analysis

Graphpad Prism 8.2.1 (Graphpad Software, Inc., La Jolla, CA, USA) was employed for data statistical analysis, which was all presented as mean ± standard error of mean (SEM). Tumor growth curve was performed with two-way ANOVA after Bonferroni correction. One-Way ANOVA and Student’s paired *t*-test analysis were performed for three or two groups’ comparisons, respectively, and non-parametric log-rank tests were used for survival rates. * indicate *P*-values ≤ 0.05 was considered significant, ** indicate *P*-values ≤ 0.01, *** indicate and *P*-values ≤ 0.001, **** indicate *P*-values ≤ 0.0001, ns indicate non-significance.

## Results

### *P. aeruginosa* Stimulation Significantly Inhibited TC-1 Growth *In Vivo*

**Figure 1:**
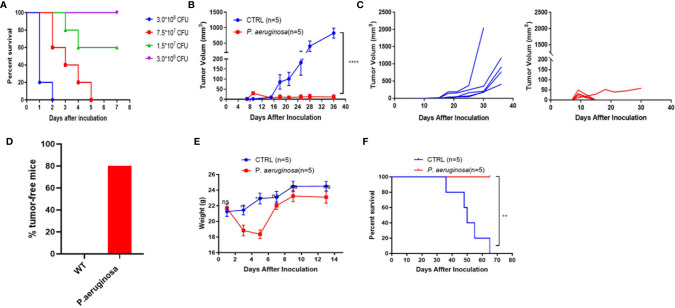
The low sublethal dose of *P. aeruginosa* co-injection inhibits tumor growth in the mouse TC-1 model. **(A)** Survival rate of mice subcutaneously challenged by a gradient of *P. aeruginosa* concentrations (n=8). Mice were continuously monitored for 7 days, which was considered the endpoint. **(B)** Tumor growth curve shown as average tumor volumes and standard error of the mean per group (n=5). **(C)** Tumor growth curve of individual mice are shown for control (n=5) and *P. aeruginosa* (n=5) co-injected groups. Numbers in the panel demonstrated tumor size and the number of mice are still alive at day 40. This experiment was repeated twice. **(D)** Tumor-free mice percentage. Tumors were grown at early stage in both group, but only one tumor-bearing in *P. aeruginosa* co-injected group to the endpoint and all mice in control group were tumor-bearing mice. **(E)** The mice body weight monitor curve. After bacteria-tumor mix injection, all mice weight was continuously monitored for 14 days. The weight of mice in *P. aeruginosa* co-injected group dropped down at early stage and recovered at day 7. **(F)** Survival curves of tumor bearing mice (n=5). All mice in control group were dead before day 70 after tumor cells incubation while none of the dead mice appeared in the *P. aeruginosa* co-injected group. Data are means ± SEM. Paired Student’s *t*-test was performed for mice body weight comparisons, Two-way ANOVA for tumor growth curve comparisons, and survival curve analysis by Log Rank (Mantel-cox) test. ** indicate *P*-values ≤ 0.01, *** indicate *P*-values ≤ 0.001, **** indicate *P*-values ≤ 0.0001, and ns indicates non-significance.

A mouse lung epithelial cell line, TC-1, which was co-transfected with HPV16 E6, E7, and Ras into C57BL/c (H-2b) cell that was broadly used as an HPV tumor model, was performed to establish a subcutaneous solid tumor model to evaluate *P. aeruginosa*-mediated anti-tumor response *in vivo*. First, the mouse sepsis model was established by subcutaneous administration with a dose-dependent bacteria injection and the mice survival rate was monitored in a week, and the survival curves indicated that none of the mice in the lowest-dose group was dead (**Figure 1A**). We chose the lowest dose of 3 × 10^6^ CFU per mouse for further analysis. TC-1 cells were co-injected with bacteria subcutaneously in the right side of the mouse, and tumor growth curve was continuously monitored (**Figure 1B**). The results of tumor volume in mice showed that the bacteria co-injection group suppressed tumor growth more significantly than that in the control group. Obviously, all tumors were growing well in the control group and only one tumor in bacteria group has a volume less than 100 mm^3^ at the observation endpoint (**Figure 1C**). These results demonstrated the robust anti-tumor response resulted from bacteria co-injection that 80% of mice in the bacteria group were tumor-free mice (**Figure 1D**). Meanwhile, we also monitored the weight of mice after tumor inoculation. After bacteria co-injection, mouse weights recovered after a slight loss of body weight (**Figure 1E**). All the mice in the bacteria co-injection group survived to the end of the experimental period, demonstrating a 100% protective efficacy, and all mice in the control group were dead in two months (**Figure 1F**). Altogether, we showed that *P. aeruginosa* co-injection suppresses tumor growth and prolongs survival *in vivo*.

### Rechallenge Tumor Cells Indicated *P. aeruginosa*-Mediated Long-Lasting Antitumor Immunity

**Figure 2:**
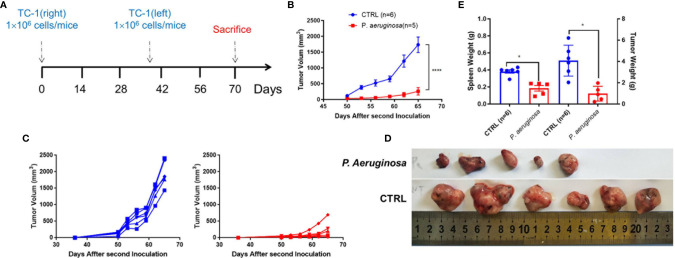
Development of long-lasting antitumor immunity against TC-1 tumor cells following *P. aeruginosa* co-injection. **(A)** Schematic diagram of the procedures for tumor rechallenge experiment. Tumor-free mice were rechallenged with 1 × 10^6^ TC-1 tumor cells again at day 40 on the other side of the primary tumor. **(B)** Rechallenged tumor growth curve shown as average tumor volumes and standard error of the mean per group (n=5–6). Statistical analysis was performed with two-way ANOVA after Bonferroni correction. **(C)** Rechallenged tumor growth curve of individual mice are shown for control (n=6) and *P. aeruginosa* (n=5) co-injected groups. Numbers in the panel demonstrated tumor size and the number of mice are still alive at day 40. This experiment was repeated twice. **(D)** Representative photographs of rechallenged tumor tissues harvested from each group. **(E)** Tumor and spleen weight of tumor-bearing mice at the end of the experiment. Data are means ± SEM. Paired Student’s *t*-test was performed for spleen and tumor weight comparisons and two-way ANOVA for tumor growth curve comparisons. * indicate *P*-values ≤ 0.05 was considered significant, **** indicate *P*-values ≤ 0.0001, and ns indicates non-significance.

We hypothesized that *P. aeruginosa*-mediated anti-tumor response could exist in a long-lasting function *in vivo*. To verify the thought, we rechallenged mice with 1 × 10^6^ TC-1 tumor cells at day 40 in the left side and monitored continuously (**Figure 2A**). The tumor growth curve showed that the rechallenged tumor growth in bacteria-treated group was significantly suppressed (**Figure 2B**). Rechallenged tumor growth curve of individual mice suggested that although tumors kept growing in both groups, the tumor growth in the bacteria-treated group was much slower than that in the control group (**Figure 2C**). At the endpoint of the experiment, all tumor-bearing mice were sacrificed, and tumors and spleen tissues were harvested. Photographs of rechallenged tumor tissues that were stripped from sacrificed mouse indicated that the rechallenged tumor volumes were smaller in the bacteria injection group than that in the control group (**Figure 2D**). In addition, the weights of tumors and spleen were also significantly lower in the bacteria group (**Figure 2E**). Consistently, these results suggested that *P. aeruginosa* mediated a robust long-lasting anti-tumor response *in vivo*.

### Immunotherapeutic Bacteria *P. aeruginosa* Activate Multiple Cell Death *In Vitro*

**Figure 3:**
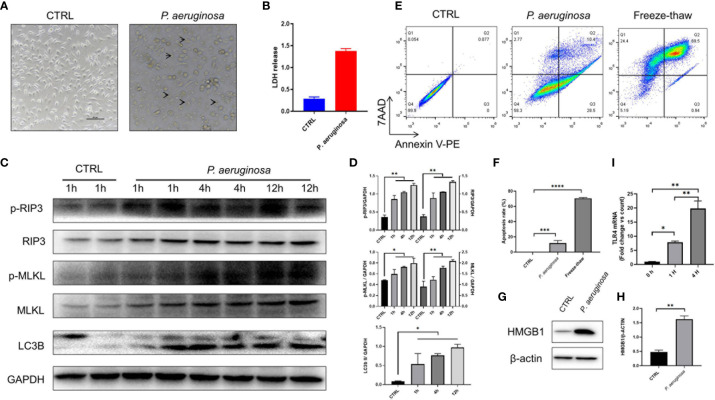
*P. aeruginosa* incubation triggered PANoptosis in TC-1 tumor cells.1 × 10^6^ tumor cells were infected with 10 MOI *P. aeruginosa* for 1 h, 4 h, and 12 h, cell culture supernatants were gently removed and tumor cells were photographed and harvested for immunoassay. **(A)** Representative photographs of tumor cells treated with or without *P. aeruginosa*. After incubation, tumor cells were swollen and lysed under the microscope. Arrow represented dying cells. **(B)** Histogram analysis of lactate dehydrogenase (LDH) release. LDH releasements are represented for tumor cell death *in vitro*. **(C)** Western blots of RIP3/pRIP3/MLKL/pMLKL and LC3B in TC-1 cells treated with *P. aeruginosa*. The WB bands intensity was quantified by image J software. **(D)** Histogram analysis of the ratio of pRIP3/GAPDH, pMLKL/GAPDH, RIP3/GAPDH, MLKL/GAPDH, LC3B II/GAPDH. **(E)** Represent FCM images of 7AAD-Annexin V-PE assay. Freeze-thawed TC-1 cells were performed as positive control. **(F)** The results of cell apoptosis assay. **(G)** Western blots of HMGB1. **(H)** Histogram analysis of HMGB1 expression. **(I)** Histogram analysis of TLR4 expression by RT-PCR in a time-dependent manner. Data are means ± SEM. Paired Student’s *t*-test was performed for two group comparisons and one-way ANOVA for three groups. * indicate *P*-values ≤ 0.05 was considered significant, ** indicate *P*-values ≤ 0.01, *** indicate *P*-values ≤ 0.001, **** indicate *P*-values ≤ 0.0001, and ns indicates non-significance.

To explore the molecular mechanisms of *P. aeruginosa* induced anti-tumor response, we co-cultured *P. aeruginosa* and TC-1 tumor cells *in vitro*. As excepted, tumor cells were dead after incubating with bacteria (**Figure 3A**), which was also confirmed by LDH release (**Figure 3B**). To examine if necrotic cell death occurred with *P. aeruginosa* incubation, we performed TC-1 cells infected with *P. aeruginosa* and examined the cell death pathways. We detected the active, phosphorylated form of RIP3 and MLKL in the lysate cell supernatants of bacteria treated tumor cells, indicating that bacteria were able to induce necrotic cell death activation. Furthermore, we observed that p-RIP3 and p-MLKL were upregulated with bacteria incubation, as well as naive RIP3 and MLKL (**Figures 3C, D**). In addition, we also detected the active LC3B in treated cell lysate and the ratio of LC3B II/GAPDH were increased, conforming that autophagic cell death is activated in response to bacterial infection (**Figures 3C, D**). To determine whether *P. aeruginosa* infection can induce apoptosis, we performed a FCM analysis by 7AAD-Annexin V-PE apoptosis kit. Notably, after bacterial incubation, the percent of apoptotic TC-1 cells was increased and the freeze-thaw tumor cells as a positive control (**Figures 3E, F**). To understand whether *P. aeruginosa* induced necrotic cell death drives immunogenic cell death (ICD) *in vitro*, we tested the release of the ICD marker HMGB1 with or without *P. aeruginosa* incubation (**Figures 3G, H**). We also found that TLR4 expression was increased in a time-dependent manner, indicating bacterial-triggered cell death *via* activated TLR4 signaling pathway (**Figure 3I**). Taken together, the results indicated *P. aeruginosa* infection triggered PANoptosis *in vitro*, including necroptosis, apoptosis, and autophagy. Furthermore, bacteria-mediated necrotic cell death is also a form of ICD *in vitro*.

### *P. aeruginosa* Co-Injection Induced Tumor Tissues Cell Death and Inhibited Proliferation

**Figure 4:**
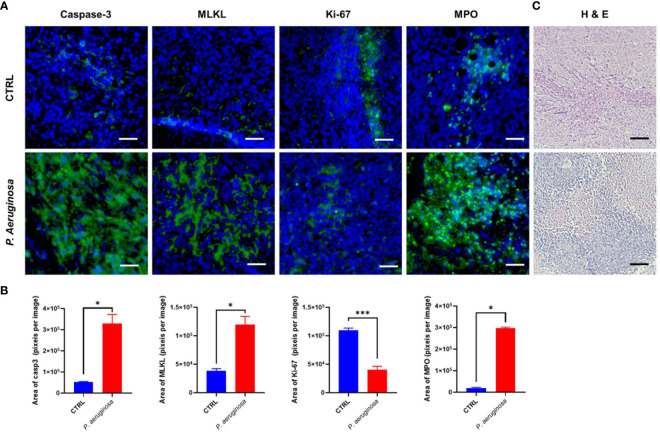
Immunofluorescence assay for the detection of cell death and proliferation in tumor tissues. **(A)** Representative images of the immunofluorescence assay for tumor tissue sections. Caspase-3 staining was identified as cell apoptosis; MLKL staining was identified as cell necroptosis; MPO staining was identified as Neutrophils (31); Ki-67 staining was identified as cell proliferation. Cell nuclei were stained with DAPI. **(B)** Quantitative analysis of FITC positive cells in tumor tissues. All experiments were repeated three times. The area of immunofluorescence intensity was quantified by image J software. **(C)** H & E staining for tumor tissue sections collected at the experimental end point. Data are means ± SEM. Paired Student’s *t*-test was performed for comparisons. * indicate *P*-values ≤ 0.05 was considered significant, *** indicate *P*-values ≤ 0.001, and ns indicates non-significance.

*P. aeruginosa* co-injection caused long-lasting tumor suppression in mice and induced tumor cell death *in vitro*. In line with these findings, we observed the hallmarks of cell death expression in tumor tissues; compared to the control group, the immunofluorescence results show that caspase-3, MPO, and MLKL expression in tumor tissues after bacteria treatment were significantly increased (**Figure 4A**). Quantification analysis confirmed that bacteria co-injection also induced tumor cell death in tissues (**Figure 4B**). Consistent with this data, we also detected Ki-67 expression levels in tumor tissues, which was considered as a hallmark of cell proliferation. The immunofluorescence results show that Ki-67 was reduced in the bacteria group (**Figure 4A**). Statistical analysis confirmed that bacterial infection induced antitumor response results in inhibition of tumor cell proliferation (**Figure 4B**). Additionally, H & E staining analysis shows that more necrotic areas were observed and more inflammatory cells were infiltrated, respectively (**Figure 4C**). Taken together, our results indicated that *P. aeruginosa* infection induced anti-tumor response can promote tumor tissues cell death and suppress tumor cells proliferation.

### Pre-Injection With *P. aeruginosa*-Tumor Cell Mixture Reshape the Tumor Microenvironment

**Figure 5:**
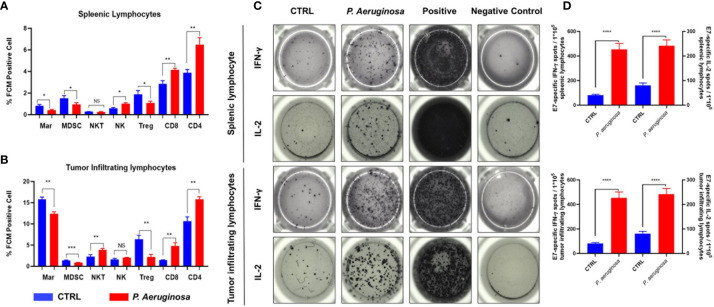
*P*. *aeruginosa* mediated anti-tumor response remodeled systematic and tumor microenvironment immune cell profiles and promoted lymphocytes activation. Histogram analysis of immune cell profiles in **(A)** splenic lymphocytes and **(B)** tumor infiltrating lymphocytes by FCM. CD3^+^CD4^+^ staining cells were identified as CD4 T cells; CD3^+^CD8^+^ staining cells were identified as CD8 T cells; CD4^+^CD25^+^ Foxp3^+^ staining cells were identified as Treg cells; CD3^-^NK1.1^+^ staining cells were identified as NK cells; CD3^+^NK1.1^+^ staining cells were identified as NK T cells; CD11b^+^Gr-1^+^ staining cells were identified as MDSC cells; CD11b^+^F4/80^+^ staining cells were identified as macrophages cells; **(C)** representative images of ELISPOT assay. Indicated 1 × 10^5^ splenic lymphocytes and tumor infiltrating lymphocytes were stimulated with 2 ug/ml E7 specific peptides. Spots were represented as activated lymphocytes, PMA was performed as positive control. **(D)** Quantitative analysis of antigen specific IFN-γ- and IL-2-expressing lymphocytes detected by ELISPOT. Data are means ± SEM. Paired Student’s *t*-test was performed for comparisons. * indicate *P*-values ≤ 0.05 was considered significant, ** indicate *P*-values ≤ 0.01, *** indicate *P*-values ≤ 0.001, **** indicate *P*-values ≤ 0.0001, and ns indicates non-significance.

To better understand the function of systemic and tumor microenvironment for antitumor immunity, we assessed the immune cell composition in tumor and splenic lymphocytes. Herein, we used the HPV 16 E7_49-57_ peptide as tumor specific antigen for stimulation, and the immunocytes profiles of tumor-infiltrating and splenic lymphocytes were analyzed *via* flow cytometry (FCM) assay (**Figures 5A, B**). FCM analysis showed that percentages of CD4 and CD8 positive T cells were increased in the splenic and tumor lymphocytes. In line with this result, we found that tumor infiltrated CD3^-^NK1.1^+^ nature killer cells (NK) exhibited an identical tendency but there was no significant difference in the number of tumor infiltrated CD3^+^NK1.1^+^ nature killer T cells (NKT) in microenvironment. In addition, the percentage of CD4^+^CD25^+^FoxP3^+^ labeled Treg cells were decreased in the bacteria-treated group. Analysis of the myeloid compartment showed decreased fractions of CD11b^+^Gr-1^+^ labeled MDSCs and CD11b^+^F4/80^+^ M2 macrophages (Mar) in the control group than that in the bacteria group. Meanwhile, the ELISPOT results indicated that IFN-γ and IL-2 produced splenic and tumor infiltrating lymphocytes were significantly increased (**Figure 5C**). Quantification results showed that the number of plots was increased and tumor antigen-specific cellular immune response in tumor-bearing mice were promoted (**Figure 5D**). In summary, our results suggest that *P. aeruginosa* infection increased antitumor lymphocytes accumulation and reduced immune-suppressive myeloid cell frequencies during tumor progression, thus inducing remarkable anti-tumor response and promoting antitumor cytokine-producing lymphocytes.

### *P. aeruginosa*-Mediated Immunogenic Cell Death Promotes DC Maturation

**Figure 6:**
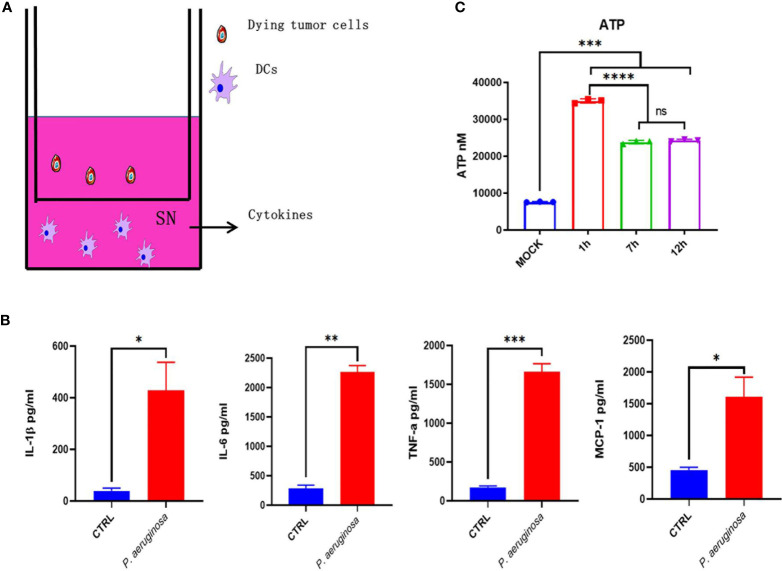
Dying tumor cells trigger DC maturation. **(A)** Schematic diagram of incubation of DC2.4 with dying tumor cells. **(B)** The concentration of cytokines with IL-1β, IL-6, TNF-α, and MCP-1 in DC supernatant after treatment. **(C)** Histogram analysis of ATP releasement. One-way ANOVA and paired Student’s *t*-test was performed for comparisons. * indicate *P*-values ≤ 0.05 was considered significant, ** indicate *P*-values ≤ 0.01, and *** indicate *P*-values ≤ 0.001, **** indicate *P*-value ≤ 0.0001.

To investigate whether bacteria-mediated tumor cell immunogenic cell death can active DCs’ function, we incubated DCs with bacteria treated- and none-treated tumor cells *in vitro*, respectively, and then the DC maturation and cytokines was assessed. We detected the cytokines release for the identification of DCs functional maturation. As expected, DCs responded to bacteria treated tumor cells with significant increase in cytokines secretion, including IL-1β, IL-6, MCP-1, and TNF-α (**Figure 6**). In line with these results, we also detected the ATP release of TC-1 cells under bacterial treatment. We found that ATP were sharply increased in the first one hour and then maintained a high level after a slight decrease (**Figure 1C**). Collectively, these results indicate bacteria treated tumor cells induced immunogenic cell death that triggered migration and maturation of DCs.

## Discussion

Herein, we have applied the immunotherapeutic role of bacteria *P. aeruginosa* in cancer therapy in a TC-1 grafted tumor model, which empowered local therapeutic and promoted a long-lasting anti-tumor immunity. Furthermore, we characterized a single, extremely low dose of bacteria incubation that triggered tumor cell PANoptosis not only in primary tumor sites but also suppressed distant untreated tumors. Our data suggest that treating with bacteria *P. aeruginosa* reshaped the systemic- and tumor-microenvironment, matured DCs, which result in a robust, durable anti-tumor immune response.

Bacteria has shown its anti-tumor propensity in the long history of cancer immunotherapy on the basis of these demonstrated theories: (1) The colonization and growth of bacteria consume oxygen and nutrition in tumor tissues; (2) Bacteria and their metabolites directly promote cell death; (3) The reconstructed tumor microenvironment enhances the capability to kill tumor cells and suppress tumor growth by immune cells. Although surgery is the most used clinical treatment, radiotherapy, chemotherapy, and immune checkpoint therapy also become prevailing for cancer treatment today. However, the high cost, drug resistance, and side-effects of these treatments narrow the clinical therapeutic applicability. Here, we used a single, extremely low dose of bacteria *P. aeruginosa* as a novel “oncolytic bacteria” to induce tumor necrotic cell death, and thus promote tumor regression at the primary sites and also inhibiting rechallenged tumor growth in the distant sites (**Figures 1** and **2**). Meanwhile, the theory of oncolytic bacteria has already been proved by other groups (32, 33). Recently, the SLC delivery system in the safe and widely applied programmed probiotics Ecoil Niddle 1917 that constitute produced nanobody antagonist of PD-L1, CTLA-4 (33), or CD47^5^ combined with a controllable lysing mechanism, a minimized of toxicities in tumor tissues. The application of oncolytic bacteria successfully stimulated antitumor immunity, promoted tumor regression directly within the tumor microenvironment in multiple syngeneic mouse models for inhibiting primary tumor growth and distant metastasis colonization, indicating the application prospect of oncolytic bacteria in the further treatment.

Oncolytic bacteria is first identified by Danino T’ group, which possesses some natural advantages for cancer immunotherapeutic: (1) targeting effects, with the growth of tumor tissues, the immune-suppressed, and hypoxia TME provides the hotbed for facultative anaerobic bacteria and anaerobic bacteria colonization; (2) modifiable genome, the bacteria hold a huge genome that could be easily and widely applied for gene editing; (3) easy to grow, multiple cell culture powders have been developed for bacteria growth; (4) cheap, the costs for bacteria growth are cheap; (5) natural adjuvant effects, numerous evidence demonstrate that the bacteria ghosts, flagella, CPG, RNA, and DNA are ligands for TLR or NLR, which activate a natural immune pathway.

We employed *P. aeruginosa* to identify its potential role in antitumor response as a novel oncolytic bacterial. First, we defined that *P. aeruginosa* directly trigger tumor cell death in a necrotic-like death by increasing the activate form of phosphorylation- RIP3 and MLKL expression in tumor cells (**Figure 3**). Pervious works identified that expression of RIP1/3 and MLKL was not only necessary to the necroptosis pathway but also contributes to the immunogenic cell death in TC-1 tumor cells (29). The expression of RIP3 was found down-regulated over half of the test cancer cell lines, suggesting tumor cells evade necrotic cell death, which is an important immune escape mechanism (34, 35). Moreover, the hypoxia TME and promoted methylation also contribute in RIP3 regulation for antitumor response (34). In addition, the expression of MLKL was also found decreased in multiple tumor tissues including ovarian carcinoma, gastric cancer, colon cancers, and so on (36–38). Then, we found that bacteria also triggered high levels of caspase-3-dependent apoptosis and other cell death like necroptosis and NETosis in tumor tissues; in the meantime, cell proliferation was also decreased according to Ki67 detection (**Figure 4**). Our previous works indicated that no matter HBV virus-like particles- (VLPs) or bacterial outer membrane vesicles (OMVs)-based tumor vaccines, which directly deliver cytokines or tumor associated-antigens (TAAs), can suppress tumor growth and reconstituted the immune-suppressed tumor microenvironment (39–44). At last, our results also indicated oncolytic bacteria *P. aeruginosa* produced durable tumor regression and reshaped the systemic and tumor-infiltrating immune cells profile changes, especially IFN-γ- and IL-2-producing lymphocytes (**Figure 5**). More importantly, we found that NK and/or NKT cells were also remodeled by bacteria treatment. In summary, *P. aeruginosa* is proved to induce TC-1 tumor cell death efficiently as well as educate the host immune system *in vitro* and *in vivo*. Future modified and attenuated P. aeruginosa could be used in combination therapy such as surgery, chemotherapy, or radiotherapy for treatment of multiple-malignant tumors. For these tumor tissues that are difficult to remove surgically or have to undergo palliative resection, *P. aeruginosa* has potential to effectively suppress metastasis *via* direct intra-tumor injection (45) or spray-on metastases and lymphatic areas.

As one of the most important specific antigen presentation cells in the immune system, DCs perform robust antigen presentation capability. There are many advantages for DCs acting as APCs: (1) overexpression of MHC-II on the cell surface; (2) upregulated expression of antigen uptake and specific transport receptors; (3) effective antigen uptake, processing, and migration to T cell niches; (4) activate naïve T cells and promote T cell proliferation and differentiation; (5) enhance antigen presentation efficiency and activate T cell function. Notably, the expression of chemokine profile change is related to DCs differentiation, maturation, and realization of antigen presentation, which is the most important feature of DCs. It has been reported that chemokine CCR1, CCR2, CCR5, CXCR1, and CXCR2 are expressed on immature DCs, while CCR7 and CXCR4 are expressed on mature DCs. Excessive bacterial infection will definitely cause macrophages and/or DC cell death, but a moderate amount of bacterial infection can effectively activate DCs and help antigens presentation. So in **Figure 1** we first evaluated the dose of *P. aeruginosa in vivo*, and we choose the lowest infection dose that causes minimal damage. Herein, oncolytic *P. aeruginosa* mediated dying tumor cell co-cultured with DC2.4 enhance cytokines release, representing the maturation of DC cells. The expression of MCP-1, which is a ligand of CCR2, located on DC membranes was upregulated in epithelial cell-derived tumor cells. Mature DCs may be caused by calcium influx and/or PI3K-MAPK single pathway activating, which needs be examined further. After stimulating with E7 peptides, IL-2-producing lymphocytes were increased (**Figure 5B**), which also could promote DC differentiation and regulate the transition of Th0 cells to Th1 cells. IFN-γ-producing lymphocytes (**Figure 5B**) from CTL, Th1, and/or NK cells can also directly promote tumor cell death in TME. The dying tumor cells can release HMGB1 (**Figures 3G, H**) that matured DCs through TLR-4-dependent manner. After matured DCs migrate to regional lymph nodes, tumor associated-antigens will be presented for stimulating T cell immune response. Overall, in our study, we found that the dying tumor cells significantly activated DC maturation.

## Conclusion

In this study, we demonstrated *P. aeruginosa* was utilized as an efficient oncolytic bacterial anticancer therapy, which can modulate TME through activating tumor cell PANoptosis and DC maturation. Given the potency and excellent safety at the clinical level, our study further suggested a promising bacteria treatment within the anti-tumor immunotherapy strategies.

## Data Availability Statement

The original contributions presented in the study are included in the article/supplementary material. Further inquiries can be directed to the corresponding author.

## Ethics Statement

The animal study was reviewed and approved by The Ethics Committee of Animal Care and Welfare of IMBCAMS and PUMC (Permit Number: SYXK(dian)2010-0007.

## Author Contributions

J-LQ and Y-BM conceptualized, designed, and supervised this study and take responsibility for data integrity and accuracy of analysis. J-RH and J-LQ acquired, analyzed, and interpreted the data. S-MJ, XY, and CL provided materials and technical support. J-RH, S-MJ, and J-LQ wrote this manuscript. Y-BM revised and edited the manuscript. All authors contributed to the article and approved the submitted version.

## Funding

This work was financially supported by the CAMS initiative for innovative medicine (grant number 2016-12M-1-019), the Fundamental Research Funds for the Central Universities of China (grant number 3332019162), and the funds for IMBCAMS PhD Innovation (grant number 2018018001) and the Foundation for Studying Abroad from the China Scholarship Council (grant number 201906210477 and 201808110121).

## Conflict of Interest

The authors declare that the research was conducted in the absence of any commercial or financial relationships that could be construed as a potential conflict of interest.

## Abbreviations

TME, Tumor microenvironment; RIP3, Receptor Interacting Serine/Threonine Kinase 3; MLKL, Mixed Lineage Kinase Domain Like Pseudokinase; HMGB1, High Mobility Group Box 1; PA-MSHA, *Pseudomonas aeruginosa*–mannose-sensitive hemagglutinin; CB-CIK, Cord-blood cytokine-induced killer; TLR, Toll-like receptor; DC, Dendritic cells; NK, Nature killer cells; NKT, Nature killer T cells; TAM, Tumor associated macrophages; MDSC, Myeloid-derived suppressor cells; PDA, Pancreatic ductal adenocarcinoma; CAC, Colitis-associated cancer; ICD, Immunogenic cell death; DAMPs, Danger-associated molecular patterns; LDH, Lactate dehydrogenase; MPO, Myeloperoxidase; FCM, Flow cytometry; OMVs, outer membrane vesicles; VLP, virus-like particles; TAAs, Tumor associated-antigens.

## References

[1] Elinav E Garrett WS Trinchieri G. The cancer microbiome. Nat Rev Cancer (2019) 19:371–6. 10.1038/s41568-019-0155-3 PMC6700740 31186547

[2] Zitvogel L Ayyoub M Routy B Kroemer G. Microbiome and Anticancer Immunosurveillance. Cell (2016) 165:276–87. 10.1016/j.cell.2016.03.001 27058662

[3] Pope JL Tomkovich S Yang Y Jobin C. Microbiota as a mediator of cancer progression and therapy. Trans Res J Lab Clin Med (2017) 179:139–54. 10.1016/j.trsl.2016.07.021 PMC5674984 27554797

[4] Decker WK da Silva RF Sanabria MH Angelo LS Guimarães F Burt BM. Cancer Immunotherapy: Historical Perspective of a Clinical Revolution and Emerging Preclinical Animal Models. Front Immunol (2017) 8:829. 10.3389/fimmu.2017.00829 28824608 PMC5539135

[5] Chowdhury S Castro S Coker C Hinchliffe TE Arpaia N. Programmable bacteria induce durable tumor regression and systemic antitumor immunity. Nat Med (2019) 25:1057–63. 10.1038/s41591-019-0498-z PMC6688650 31270504

[6] Sommariva M Le Noci V. The lung microbiota: role in maintaining pulmonary immune homeostasis and its implications in cancer development and therapy. Cell Mol Life Sci (2020) 77:2739–49. 10.1007/s00018-020-03452-8 PMC7326824 31974656

[7] Carbone C Piro G Di Noia V D’Argento E Vita E Ferrera MG. Lung and Gut Microbiota as Potential Hidden Driver of Immunotherapy Efficacy in Lung Cancer. Mediators Inflamm (2019) 2019:7652014. 10.1155/2019/7652014 31827379 PMC6885300

[8] Liu C Qi J Shan B Gao R Gao F Xie H. Pretreatment with cathelicidin-BF ameliorates Pseudomonas aeruginosa pneumonia in mice by enhancing NETosis and the autophagy of recruited neutrophils and macrophages. Int Immunopharmacol (2018) 65:382–91. 10.1016/j.intimp.2018.10.030 30380513

[9] Tanamoto K Abe C Homma JY Kojima Y. Regions of the lipopolysaccharide of Pseudomonas aeruginosa essential for antitumor and interferon-inducing activities. Eur J Biochem (1979) 97:623–9. 10.1111/j.1432-1033.1979.tb13152.x 111929

[10] Zhang Z Wang LP Zhao XL Wang F Huang L Wang M. Pseudomonas aeruginosa injection enhanced antitumor cytotoxicity of cytokine-induced killer cells derived from cord blood. Biomed Pharmacother = Biomed Pharmacother (2014) 68:1057–63. 10.1016/j.biopha.2014.10.024 25465152

[11] Zhang M Luo F Zhang Y Wang L Lin W Yang M. Pseudomonas aeruginosa mannose-sensitive hemagglutinin promotes T-cell response via toll-like receptor 4-mediated dendritic cells to slow tumor progression in mice. J Pharmacol Exp Ther (2014) 349:279–87. 10.1124/jpet.113.212316 24623801

[12] Wei Y Liu D Jin X Gao P Wang Q Zhang J. PA-MSHA inhibits the growth of doxorubicin-resistant MCF-7/ADR human breast cancer cells by downregulating Nrf2/p62. Cancer Med (2016) 5:3520–31. 10.1002/cam4.938 PMC5224842 27758045

[13] Li T Dong ZR Guo ZY Wang CH Zhi XT Zhou JW. Mannose-mediated inhibitory effects of PA-MSHA on invasion and metastasis of hepatocellular carcinoma via EGFR/Akt/IkappaBbeta/NF-kappaB pathway. Liver Int Off J Int Assoc Study Liver (2015) 35:1416–29. 10.1111/liv.12644 25066210

[14] Xu WH Liu ZB Hou YF Hong Q Hu DL Shao ZM. Inhibition of autophagy enhances the cytotoxic effect of PA-MSHA in breast cancer. BMC Cancer (2014) 14:273. 10.1186/1471-2407-14-273 24745346 PMC4000616

[15] Cheng X Wang B Jin Z Ma D Yang W Zhao R. Pseudomonas aeruginosa-mannose-sensitive hemagglutinin inhibits pancreatic cancer cell proliferation and induces apoptosis via the EGFR pathway and caspase signaling. Oncotarget (2016) 7:77916–25. 10.18632/oncotarget.12844 PMC5363631 27788491

[16] Sun L Wang H Wang Z He S Chen S Liao D. Mixed lineage kinase domain-like protein mediates necrosis signaling downstream of RIP3 kinase. Cell (2012) 148:213–27. 10.1016/j.cell.2011.11.031 22265413

[17] Ruan ZH Xu ZX Zhou XY Zhang X Shang L. Implications of Necroptosis for Cardiovascular Diseases. Curr Med Sci (2019) 39:513–22. 10.1007/s11596-019-2067-6 31346984

[18] Kitur K Parker D Nieto P Ahn DS Cohen TS Chung S. Toxin-induced necroptosis is a major mechanism of Staphylococcus aureus lung damage. PloS Pathog (2015) 11:e1004820. 10.1371/journal.ppat.1004820 25880560 PMC4399879

[19] Huang Z Wu SQ Liang Y Zhou X Chen W Li L. RIP1/RIP3 binding to HSV-1 ICP6 initiates necroptosis to restrict virus propagation in mice. Cell Host Microbe (2015) 17:229–42. 10.1016/j.chom.2015.01.002 25674982

[20] Wang Y Zhao M He S Luo Y Zhao Y Cheng J. Necroptosis regulates tumor repopulation after radiotherapy via RIP1/RIP3/MLKL/JNK/IL8 pathway. J Exp Clin Cancer Res CR (2019) 38:461. 10.1186/s13046-019-1423-5 31706322 PMC6842489

[21] Wen SH Lin LN Wu HJ Yu L Lin L Zhu LL. TNF-alpha increases Staphylococcus aureus-induced death of human alveolar epithelial cell line A549 associated with RIP3-mediated necroptosis. Life Sci (2018) 195:81–6. 10.1016/j.lfs.2018.01.008 29330116

[22] Seifert L Werba G Tiwari S Giao Ly NN Alothman S Alqunaibit D. The necrosome promotes pancreatic oncogenesis via CXCL1 and Mincle-induced immune suppression. Nature (2016) 532:245–9. 10.1038/nature17403 PMC4833566 27049944

[23] Liu ZY Zheng M Li YM Fan XY Wang JC Li ZC. RIP3 promotes colitis-associated colorectal cancer by controlling tumor cell proliferation and CXCL1-induced immune suppression. Theranostics (2019) 9:3659–73. 10.7150/thno.32126 PMC6587173 31281505

[24] Zhao Q Yu X Li M Liu Y Han Y Zhang X. MLKL attenuates colon inflammation and colitis-tumorigenesis via suppression of inflammatory responses. Cancer Lett (2019) 459:100–11. 10.1016/j.canlet.2019.05.034 31158430

[25] Van Hoecke L Van Lint S. Treatment with mRNA coding for the necroptosis mediator MLKL induces antitumor immunity directed against neo-epitopes. Nat Commen (2018) 9:3417. 10.1038/s41467-018-05979-8 PMC6109072 30143632

[26] Van Hoecke L Saelens X. Therapeutic anti-tumor immunity directed against neo-epitopes by intratumor delivery of mRNA encoding MLKL. Cell Stress (2018) 2:279–81. 10.15698/cst2018.10.160 PMC6551638 31225452

[27] Obeid M Tesniere A Ghiringhelli F Fimia GM Apetoh L Perfettini JL. Calreticulin exposure dictates the immunogenicity of cancer cell death. Nat Med (2007) 13:54–61. 10.1038/nm1523 17187072

[28] Kawano M Tanaka K Itonaga I Iwasaki T Miyazaki M Ikeda S. Dendritic cells combined with doxorubicin induces immunogenic cell death and exhibits antitumor effects for osteosarcoma. Oncol Lett (2016) 11:2169–75. 10.3892/ol.2016.4175 PMC4774596 26998143

[29] Yang H Ma Y Chen G Zhou H Yamazaki T Klein C. Contribution of RIP3 and MLKL to immunogenic cell death signaling in cancer chemotherapy. Oncoimmunology (2016) 5:e1149673. 10.1080/2162402x.2016.1149673 27471616 PMC4938314

[30] Apetoh L Ghiringhelli F Tesniere A Obeid M Ortiz C Criollo A. Toll-like receptor 4-dependent contribution of the immune system to anticancer chemotherapy and radiotherapy. Nat Med (2007) 13:1050–9. 10.1038/nm1622 17704786

[31] Azzouz D Khan MA Sweezey N Palaniyar N. Two-in-one: UV radiation simultaneously induces apoptosis and NETosis. Cell Death Discovery (2018) 4:51. 10.1038/s41420-018-0048-3 PMC5919968 29736268

[32] Chen J Danino T Prindle A Skalak M Selimkhanov J Allen K. Modulation of Salmonella Tumor-Colonization and Intratumoral Anti-angiogenesis by Triptolide and Its Mechanism. Theranostics (2017) 7:2250–60. 10.7150/thno.18816 PMC5505057 28740548

[33] Din MO Morgan MJ Lee DG Kim WJ Yoon JH. Synchronized cycles of bacterial lysis for in vivo delivery. Nature (2016) 536:81–5. 10.1038/nature18930 PMC5048415 27437587

[34] Koo GB Wang J Schilling R Horn S Harris PA Bertin J. Methylation-dependent loss of RIP3 expression in cancer represses programmed necrosis in response to chemotherapeutics. Cell Res (2015) 25:707–25. 10.1038/cr.2015.56 PMC4456623 25952668

[35] Geserick P Yu W Shen L Huang T. Absence of RIPK3 predicts necroptosis resistance in malignant melanoma. Cell Death Dis (2015) 6:e1884. 10.1038/cddis.2015.240 26355347 PMC4650439

[36] Sun W Yu W Shen L Huang T. MLKL is a potential prognostic marker in gastric cancer. Oncol Lett (2019) 18:3830–6. 10.3892/ol.2019.10687 PMC6732949 31516595

[37] He L Peng K Liu Y Xiong J Zhu FF. Low expression of mixed lineage kinase domain-like protein is associated with poor prognosis in ovarian cancer patients. OncoTargets Ther (2013) 6:1539–43. 10.2147/ott.s52805 PMC3817086 24204164

[38] Li X Sun P Xie H Huang W Qi J Ma J. Association of Mixed Lineage Kinase Domain-Like Protein Expression With Prognosis in Patients With Colon Cancer. Technol Cancer Res Treat (2017) 16:428–34. 10.1177/1533034616655909 PMC5616063 27432118

[39] Shu C Shu C Hua L Zhao Y Xie H Qi J. Virus-Like Particles Presenting the FGF-2 Protein or Identified Antigenic Peptides Promoted Antitumor Immune Responses in Mice. Int J Nanomed (2020) 15:1983–96. 10.2147/ijn.s237182 PMC7146011 32308382

[40] Huang W Liu H Chu X Sun P Huang W Liu C. Modified bacterial outer membrane vesicles induce autoantibodies for tumor therapy. Acta Biomater (2020) 100:316–25. 10.1016/j.actbio.2020.03.030 32251780

[41] Feng X Li Y Huang W Feng X Sun P Yao Y. Recombinant virus-like particles presenting IL-33 successfully modify the tumor microenvironment and facilitate antitumor immunity in a model of breast cancer. Acta Biomater (2019) 100:316–25. 10.1016/j.actbio.2019.09.024 31542504

[42] Chu X Li Y Long Q Xia Y Yao Y Sun W. Combined immunization against TGF-beta1 enhances HPV16 E7-specific vaccine-elicited antitumour immunity in mice with grafted TC-1 tumours. Artif Cells Nanomed Biotechnol (2018) 46:1199–209. 10.1080/21691401.2018.1482306 29929402

[43] Chu X Huang W Li K Yao Y Yang X Bai H. Chimeric HBcAg virus-like particles presenting a HPV 16 E7 epitope significantly suppressed tumor progression through preventive or therapeutic immunization in a TC-1-grafted mouse model. Int J Nanomed (2016) 11:2417–29. 10.2147/ijn.s102467 PMC4892837 27313455

[44] Wang S Zhang L Janku F Collins A Bai RY Staedtke V. Engineered outer membrane vesicle is potent to elicit HPV16E7-specific cellular immunity in a mouse model of TC-1 graft tumor. Int J Nanomed (2017) 12:6813–25. 10.2147/ijn.s143264 PMC5602458 28979120

[45] Roberts NJ Khan MA Sweezey N Palaniyar N. Intratumoral injection of Clostridium novyi-NT spores induces antitumor responses. (2018) 4(1):51. 10.1038/s41420-018-0048-3 PMC4399712 25122639

